# Supplementary material for: No Impact of Smoking Status on Breast Cancer Tumor Infiltrating Lymphocytes, Response to Neoadjuvant Chemotherapy and Prognosis
Source: Cancers (Basel). 2020 Oct 12;12(10):2943. doi: 10.3390/cancers12102943 (PMC7601636; doi:10.3390/cancers12102943)
Supplement: Supplementary file 1 [file cancers-12-02943-s001.pdf]

Supplementary Material: No Impact of Smoking Status on Breast Cancer Tumor Infiltrating Lymphocytes, Response to Neoadjuvant Chemotherapy and Prognosis

Vanille Simon, Lucie Laot, Enora Laas, Sonia Rozette, Julien Guerin, Thomas Balezeau, Marion Nicolas, Jean-Yves Pierga, Florence Coussy, Marick Laé, Diane De Croze, Beatriz Grandal, Judith Abecassis, Elise Dumas, Florence Lerebours, Fabien Reyal and Anne-Sophie Hamy

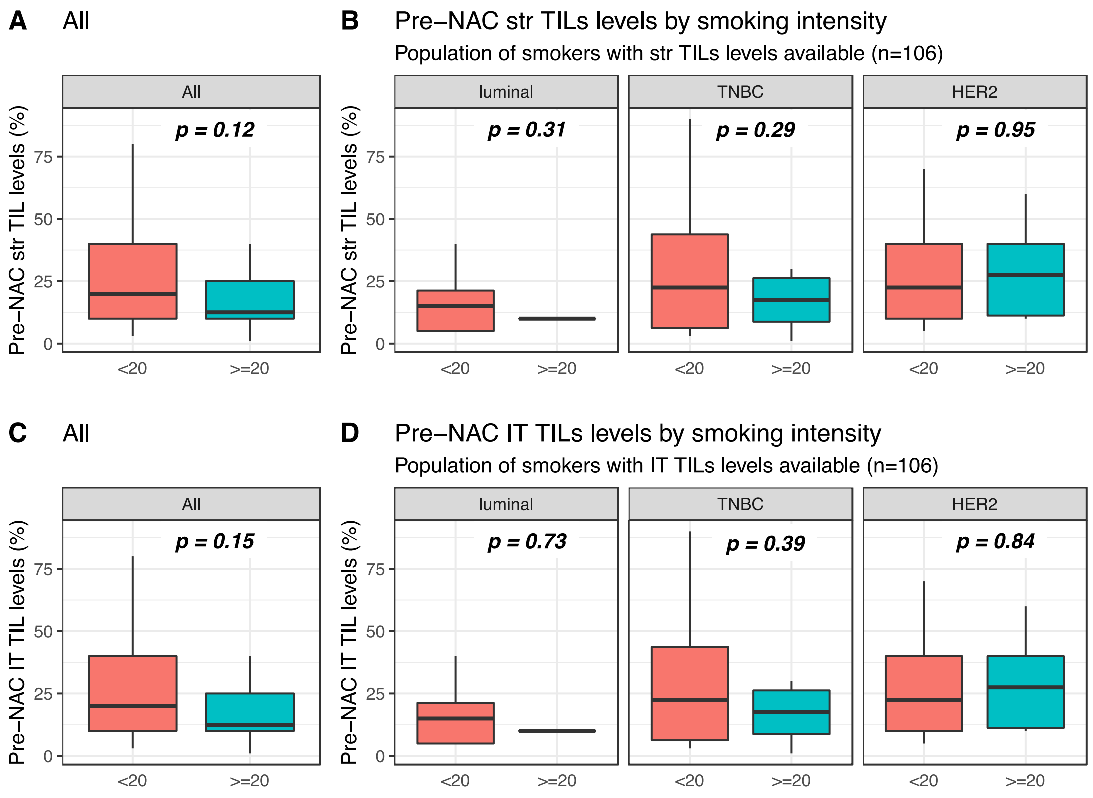

Figure S1. Pre-NAC TIL levels at BC diagnosis according to smoking amount (< 20 pack- years or ≥ 20 pack-years): Pre-NAC str TIL levels in the whole population (**A**), Pre-NAC str TIL levels by BC subtype (**B**), Pre-NAC IT TIL levels in the whole population (**C**), Pre-NAC IT TIL levels by BC subtype (D).

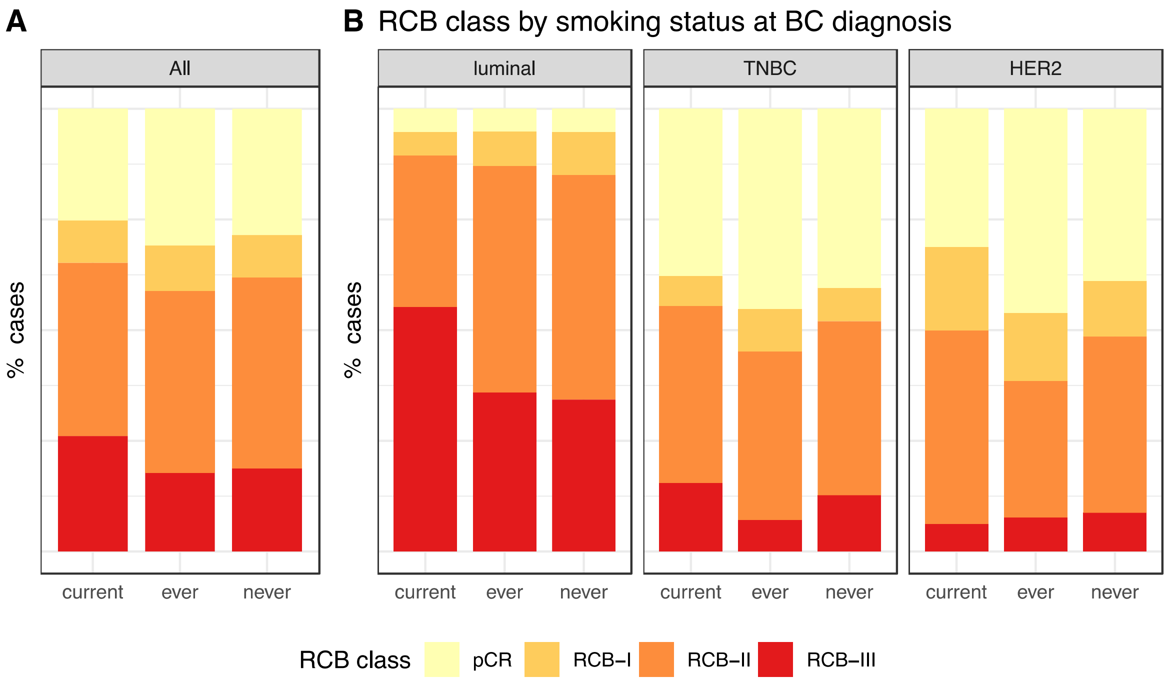

Figure S2. RCB class at NAC completion, according to smoking status, in the whole population (**A**) and by BC subtype (**B**).

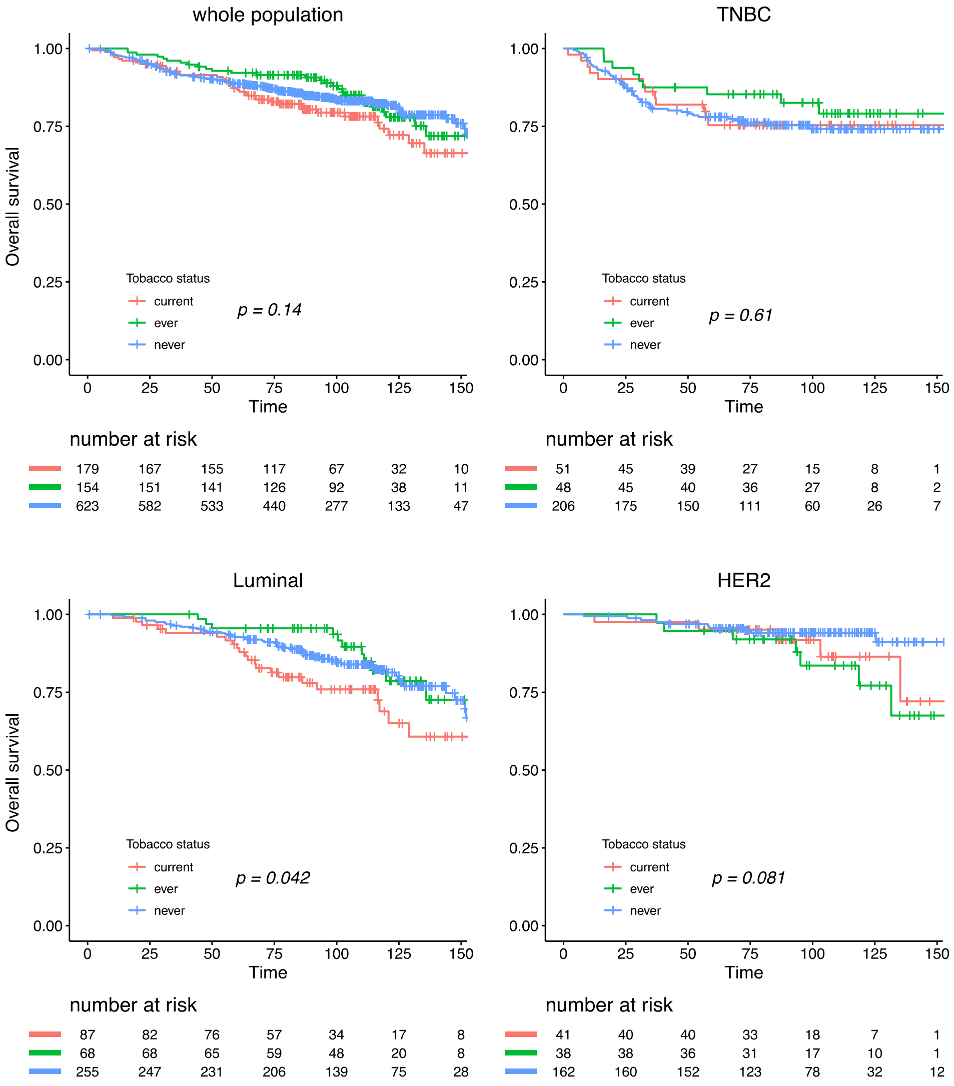

Figure S3. Overall survival according to smoking status in the whole population and by BC subtype.

Table S1. Univariate and multivariate analysis on overall survival in the luminal BC population. Abbreviations: pCR = pathological Complete Response; NAC = Neoadjuvant Chemotherapy; RCB=Residual Cancer Burden; LVI = Lympho-Vascular Involvement; str = stromal; IT = intratumoral. In the case of nonnormal continuous variables *

**Luminal**									
Characteristics			Univariate				Multivariate		
Variable	Number	Events	HR	CI	*p* *	*p*	HR	CI	*p*
Pre-NAC parameters									
Age (years)			1,004	[0.9891–1.019]		0,892			
Menopausal status									
Pre-menopausal	281	48	1			0,055			
Post-menopausal	125	31	1,56	[0.99–2.45]					
BMI class									
19≤BMI≤25	221	39	1			0,111			
<19	31	7	1,43	[0.64–3.2]					
]25-30]	101	17	0,87	[0.49–1.54]					
>30	57	18	1,8	[1.03–3.15]					
Tumor size									
T1	15	4	1			0,207			
T2	281	49	0,58	[0.21–1.62]					
T3	114	28	0,85	[0.3–2.43]					
Clinical nodal status									
N0	175	28	1			0,039	1		
N1-N2-N3	234	53	1,63	[1.02–2.58]	0,039		1,63	[1.02–2.58]	0,039
Mitotic Index									
≤22	153	30	1			0,195			
>22	57	14	1,53	[0.8–2.92]					
Histology									
NST	199	41	1			0,778			
others	23	5	1,14	[0.45–2.9]					
Grade									
I-II	245	47	1			0,495			
III	150	31	1,17	[0.74–1.85]					
ki67									
<20	86	17	1			0,628			
≥20	107	23	1,17	[0.62–2.19]					
PR status									
negative	43	12	1			0,363			
positive	161	29	0,73	[0.37–1.44]					

NAC regimen						
AC	61	12	1			0,182
AC-Taxanes	301	59	1,84	[0.96–3.52]		
Others	48	10	1,8	[0.77–4.21]		
str TILs			0,99	[0.979–0.999]		0,705
IT TILs			0,97	[0.946–0.987]		0,732
Tobacco status						
current	87	23	1			0,046
ever	68	11	0,47	[0.23–0.96]	0,038	
never	255	47	0,57	[0.35–0.95]	0,03	
Post-NAC parameters						
pCR						
No pCR	382	79	1			0,203
pCR	28	2	0,4	[0.1–1.64]		
LVI						
no	197	32	1			0,055
yes	136	36	1,6	[0.99–2.57]	0,055	
str TILs			1,01	[0.995–1.02]		0,245

© 2020 by the authors. Licensee MDPI, Basel, Switzerland. This article is an open access article distributed under the terms and conditions of the Creative Commons Attribution (CC BY) license (http://creativecommons.org/licenses/by/4.0/).
